# Effect of Actin Alpha Cardiac Muscle 1 on the Proliferation and Differentiation of Bovine Myoblasts and Preadipocytes

**DOI:** 10.3390/ani11123468

**Published:** 2021-12-06

**Authors:** Anqi Li, Xiaotong Su, Yuan Tian, Guibing Song, Linsen Zan, Hongbao Wang

**Affiliations:** 1 College of Animal Science and Technology, Northwest A&F University, Yangling, Xianyang 712100, China; 13562170853@163.com (A.L.); xiaotongsu1114@163.com (X.S.); 17319512949@163.com (Y.T.); songguibing16@163.com (G.S.); zanlinsen@163.com (L.Z.); 2 National Beef Cattle Improvement Centre, Yangling, Xianyang 712100, China

**Keywords:** bovine, myoblasts, preadipocytes, *ACTC1*, proliferation, differentiation

## Abstract

**Simple Summary:**

Marbling is an important factor affecting the quality of beef. The co-culture (myoblast-preadipocytes) system was successfully established in our lab in the early stage to simulate the internal environment of marbling. Within this environment, *ACTC1* gene was a differentially expressed gene screened from the co-culture system. The gene was not expressed in monocultured adipocytes but was expressed in co-cultured adipocytes. Therefore, we hypothesize that the *ACTC1* gene plays a role in the development of bovine myoblasts and preadipocytes. In this study, we explored the effect of *ACTC1* gene on the proliferation and differentiation of bovine myoblasts and preadipocytes, aiming to discover the potential biological function of *ACTC1* gene in muscle development and fat deposition. The results showed that *ACTC1* could regulate the development of bovine myoblasts and preadipocytes, and *ACTC1* could be used as an important target for improving beef quality in the future.

**Abstract:**

Actin Alpha Cardiac Muscle 1 (ACTC1) gene is a differentially expressed gene screened through the co-culture system of myoblasts-preadipocytes. In order to study the role of this gene in the process of proliferation and differentiation of bovine myoblasts and preadipocytes, the methods of the knockdown, overexpression, and ectopic expression of *ACTC1* were used in this study. After *ACTC1* knockdown in bovine myoblasts and inducing differentiation, the sizes and numbers of myotube formation were significantly reduced compared to the control group, and myogenic marker genes—MYOD1, MYOG, MYH3, MRF4, MYF5, CKM and MEF2A—were significantly decreased (*p* < 0.05, *p* < 0.01) at both the mRNA and protein levels of myoblasts at different differentiation stages (D0, D2, D4, D6 and D8). Conversely, *ACTC1* overexpression induced the inverse result. After ectopic expression of *ACTC1* in bovine preadipocytes and induced differentiation, the number and size of lipid droplets were significantly higher than those of the control group, and the expression of adipogenic marker genes—FABP4, SCD1, PPARγ and FASN—were significantly increased (*p* < 0.05, *p* < 0.01) at the mRNA and protein levels of preadipocytes at different differentiation stages. Flow cytometry results showed that both the knockdown and overexpression of *ACTC1* inhibited the normal cell cycle of myoblasts; however, ectopic expression of *ACTC1* in adipocytes induced no significant cell cycle changes. This study is the first to explore the role of *ACTC1* in bovine myogenesis and lipogenesis and demonstrates that *ACTC1* promotes the differentiation of bovine myoblasts and preadipocytes, affecting the proliferation of myoblasts.

## 1. Introduction

Compared with other meats, beef is richer in protein, vitamins, and a variety of essential amino acids, so it is favored by the majority of consumers. Beef quality is determined by several factors, chief among them being muscle growth and development as well as intramuscular fat content [1,2]. At the cellular level, an increase in intramuscular fat content is characterized by increased proliferation in adipocytes and an increase in the number and size of lipid droplets (differentiation). As one of the most widely consumed meats in the world, improving the output and quality of beef has long been important to the food industry. Given that the growth and development of muscle and fat are directly related to the yield and quality of beef, understanding the regulatory mechanisms in muscle and adipose tissue growth and development at the molecular level may elucidate new strategies to impact beef quality and lay a foundation for the molecular breeding of beef cattle.

Actin Alpha Cardiac Muscle 1 (ACTC1) is the main actin in embryonic hearts and plays an important role in heart development [3,4,5]. Moreover, it is the most abundant transverse α-actin subtype in mature hearts and the main protein of myocardial myofilaments, making it responsible for the contractile function of the heart through the troponin system [6]. Cardiac actin gene mutations are thought to affect sarcomere contraction and lead to familial hypertrophic and idiopathic dilated cardiomyopathy, cardiac compensatory hypertrophy, and heart failure [7,8,9,10,11,12,13,14]. Furthermore, *ACTC1* is also the most important striated α-actin in fetal skeletal muscle [15,16]. Studies have shown that while *ACTC1* plays a critical role in the formation of early human skeletal muscle, ACTC1 protein expression is downregulated to a negligible level in fetal skeletal muscle after birth, although it may be expressed in regenerated muscle fibers [17,18]. In mouse models, transgene overexpression of *ACTC1* can alleviate muscle dysplasia diseases caused by a skeletal muscle α-actin (*ACTA1*) deficiency (*ACTA1* disease) [19]. Further study has found that the expression of *ACTC1* is related to myogenic regulatory factors. When it is co-expressed with MRF4, the N-terminal of MRF4 can inhibit the expression of *ACTC1* [20]. *ACTC1* has also been used as a marker of glioma invasion and prognosis [21]. One study even reported that *ACTC1* could be used as a biomarker for the diagnosis of liver metastasis and provide a new strategy for the development of anti-metastasis drugs in colon cancer patients [22].

At present, most studies on *ACTC1* have evaluated its effects on cardiac development and related diseases in model animals, such as humans and mice. There have been few studies on this gene in cattle. Of those that have been performed, some have shown that *ACTC1* gene copy number variation was related to beef production and quality [23]. Using bovine longissimus dorsi RNA-Seq technology, one study found that *ACTC1* is related to bovine muscle weight gain and feed intake [24]. In addition, a recent paper on *ACTC1* found that it may be related to the regulation of mammary gland development in Italian buffalos [25]. However, the effects of *ACTC1* on the proliferation and differentiation of bovine myoblasts and adipocytes remains uninvestigated.

Our lab has previously identified *ACTC1* as a differentially expressed gene via transcriptome sequencing technology when studying the mutual regulation of bovine myocytes and adipocytes (undergoing publication), and the role of *ACTC1* in the muscle-fat dialogue was preliminarily explored. In that study, *ACTC1* was not detected in preadipocytes cultured separately, but the presence of myoblasts in the co-culture (myoblasts-preadipocytes) system induced the expression of *ACTC1* in adipocytes. Herein, we utilize gene knockdown, overexpression, and ectopic expression to elucidate the proliferation and differentiation effects of *ACTC1* on separately cultured bovine myoblasts and preadipocytes.

## 2. Materials and Methods

### 2.1. Sample Source

All animal procedures conformed with the Regulations for the Administration of Affairs Concerning Experimental Animals (Ministry of Science and Technology, Beijing, China, 2004) and were approved by the Institutional Animal Care and Use Committee (College of Animal Science and Technology, Northwest A&F University, Yanling, Xianyang, China, No. 2013-23, 20 April 2013).

In this study, the 4-day-old Qinchuan beef cattle was used to isolate preadipocytes and myoblasts, and the cattle was born and raised at the breeding farm of the National Beef Cattle Improvement Center (Yangling, Xianyang, China).

### 2.2. Isolation, Culture, and Differentiation of Primary Bovine Myoblasts and Preadipocytes

Myoblasts were isolated according to Wang et al. [26] and digested by type Ⅱ collagenase (Gibco) and neutral protease (Sigma, Kawasaki, Japan). Collagenase II and neutral protease were used to digest Qinchuan cattle hind leg muscle tissue and then centrifuged to collect the supernatant (150 rpm/min, 5 min). The process was repeated until the muscle tissue was completely digested. All supernatants were centrifuged to obtain primary myoblasts (1000 rpm/min, 10 min). The isolated myoblasts were cultured in a complete growth medium of Dulbecco’s Modified Eagle Medium/F-12 (DMEM/F-12, Gibco, Grand Island, NY, USA) containing 20% fetal bovine serum (FBS, Gibco) and 1% penicillin/streptomycin (Hyclone, Logan, UT, USA).

The isolation and culture protocols for preadipocytes were performed using type I collagenase (Gibco) digestion method according to Meissburgeretal [27], with modifications established previously in our laboratory [28]. Collagenase I was used to digest Qinchuan cattle perirenal fat. After centrifugation, the primary preadipocytes were present in the pellet. Isolated preadipocytes were cultured in the complete growth medium of DMEM/F-12 containing 10% FBS and 1% penicillin/streptomycin.

Once the cells (myoblasts or preadipocytes) were 70% confluent, they were then cultured in growth medium supplemented with adenovirus. After 12 h, the media was replaced with growth medium. Forty-eight hours later was recorded as the 0th day (D0), and the media was replaced with the respective differentiation media. The differentiation medium for myoblasts contained DMEM/F-12, with 2% horse serum (HS, Gibco) and 1% penicillin/streptomycin. The differentiation medium for preadipocytes was prepared composed of complete medium containing isobutylmethylxanthine (IBMX, 0.5 mM, Sigma, Kawasaki, Japan), insulin (1 mg/mL, Sigma, Kawasaki, Japan), and dexamethasone (1 mM, Sigma, Saint Louis, MO, USA). The differentiation media were changed every two days, and cell changes (myoblasts-myotubes; preadipocytes-lipid droplets) on the 2nd, 4th, 6th, 8th, and 10th days (D2, D4, D6, D8, D10) were recorded successively. The size and number of lipid droplets and myotubes were observed under an Olympus IX71 microscope (OLYMPUS, Dalian, China). Each experiment was performed in triplicate.

### 2.3. Interference and Overexpression of Adenovirus Packaging

In this experiment, based on the sequence (Gene ID: 533219) of *ACTC1* gene published on NCBI, primers (Table 1) were designed and synthesized to amplify the coding DNA sequence (CDS) of *ACTC1* (see the next paragraph for PCR system). These were then sent to OBIO Technology (Shanghai) Corp., Ltd. for adenovirus packaging. The titers of ACTC1 knockdown adenovirus (AD-shRNA-ACTC1, pDKD-CMV-eGFP-U6-shRNA (ACTC1)) and control adenovirus (AD-shRNA-NC, pDKD-CMV-eGFP-U6-shRNA) were 6.32 × 10^10^ PFU/mL and 3.16 × 10^11^ PFU/mL, respectively. The titers of ACTC1 overexpression (ectopic expression) adenovirus (AD-ACTC1, pAdeno-MCMV-ACTC1-P2A-3Flag-EGFP) and control adenovirus (AD-NC, pAdeno-MCMV-3Flag-EGFP) were 1.34 × 10^11^ PFU/mL and 4.74 × 10^10^ PFU/mL, respectively.

PCR system: DNA 100 ng/μL, upstream and downstream primers 2.5 μL, dNTP Mix (2.5 mmol/L, Takara, Mountain View, CA, USA) 4 μL, 10 × PCR Buffer 5 μL (Takara), Ex Taq 0.25 μL (Takara), diluted with double distilled water to 50 μL. The reaction protocol is as follows: 95 °C 3 min, 95 °C 20 min, 55 °C 30 s, 72 °C 2 min, 30 cycles, 72 °C 5 min.

### 2.4. Optimal Adenovirus MOI Determination

Myoblasts or preadipocytes were resuscitated in a 12-well plate. When cells were at 50~70% confluency, the different concentrations of adenovirus AD-shRNA-NC (MOI = 6, 19, 32, 44), AD-shRNA-ACTC1 (MOI = 38, 51, 63, 76), AD-NC (MOI = 16, 19, 22, 27) or AD-ACTC1 (MOI = 9, 19, 29, 39) were added to the complete growth medium, and the medium was changed. After 48 h, the optimal infection concentration was determined via cell growth and green fluorescent protein (GFP) expression. RNA and protein were extracted, and *ACTC1* changes were measured by qRT-PCR and Western blot.

### 2.5. Immunofluorescence Staining

D4, D6, and D8 myocytes were stained with MYHC immunofluorescence to observe myotube changes. The cells were fixed with 4% tissue cell fixation solution (Solarbio) for 10 min; permeated with 0.2% TritonX-100 (Sigma, Kawasaki, Japan) for 20 min; incubated with phosphate-buffered saline (PBS, Hyclone) containing 10% donkey serum (Solarbio), 1% bovine serum albumin (Sangon Biotech, Shanghai, China), and 0.3 M glycine (Sigma, Kawasaki, Japan) for 1 h; incubated with MYHC antibody (mouse anti-MYHC, 1:200, Abcam, ab50967, Cambridge, NT, UK) at 4 °C overnight; then washed with PBS 3 times. Before imaging, the cells were incubated at 37 °C for 1 h with Goat Anti-Mouse IgG (H + L) (1:200, SA00013-3, Proteintech Group, Inc., Rosemont, IL, USA), the cell nucleus was stained with 4′,6-diamidino-2-phenylindole (DAPI, Gibco, Grand Island, NY, USA) for 15 min, and it was washed 3 times with PBS before being imaged on an Olympus IX71 microscope (OLYMPUS 100×, Dalian, China).

### 2.6. Oil Red O Staining

D0, D2, D4, D6, D8, and D10 preadipocytes were stained with oil red O (Sigma, Kawasaki, Japan) to observe lipid droplet changes. The oil red O solution was prepared with isopropanol (BIOFOUNT, Beijing, China) and then diluted with distilled water and filtered. The cells were washed with PBS 3 times and fixed with 4% tissue cell fixation solution for 30 min, stained with oil red O for 30 min, and washed with PBS 3 additional times. The numbers and sizes of lipid droplets were then imaged on an Olympus IX71 microscope (OLYMPUS 400×, Dalian, China). Three images were randomly collected for each observation field to calculate the area of Oil Red O staining by the software Image J (National Institute of Mental Health, Bethesda, MD, USA).

### 2.7. Flow Cytometry

When cells were at 50~60% confluency, varying concentrations of knockdown and overexpression (ectopic expression) adenoviruses were infected in myocytes and adipocytes, respectively, with each treatment performed in triplicate. After 48 h, cells were at 80–90% confluency and were digested with Trypsin-EDTA (0.25%, Gibco). Then, One Step Cell Cycle Straining Kit (MultiSciences Biotech, Hangzhou, China) was added to permeate the cells, and the cells were stained with DAPI for 30 min. Finally, the DNA content under the differing treatments was detected via flow cytometer (λ = 488 nm).

### 2.8. Real-Time Quantitative PCR

The total RNA of the cells on the 0th, 2nd, 4th, 6th, and 8th day was extracted by TRIzol reagent (Takara, Mountain View, CA, USA), and then cDNA was obtained by Prime Script RT reagent kit (Takara, Mountain View, CA, USA). In this study, glyceraldehyde-3-phosphate dehydrogenase (*GAPDH*) was used as the internal reference, and primers were designed by gene sequence on NCBI to detect the expression changes of myogenic marker genes (*MYOD1*, *MYOG*, *MYH3*, *MYF5*, *MYF6*, *MEF2A* and *CKM*) and adipogenic marker genes (*PPAR γ*, *FABP4*, *SCD1* and *FASN*) at the mRNA level. The primer sequence is shown in Table 2. Subsequently, the cDNA was used for qRT-PCR in triplicate wells by the SYBR Green Real-Time PCR Master Mix (Takara, Mountain View, CA, USA) in Bio-Rad Real-Time PCR System (Bio-Rad, Hercules, CA, USA). Then the result was calculated according to the formula 2^−ΔΔCt^. The thermal cycles used in the qRT-PCR were 40.

### 2.9. Western Blot

A protein extraction kit (Solarbio Company, Beijing, China) was used to extract the proteins on days 2, 4, 6 and 8 of adipocyte and myocyte differentiation, and the protein expression of the corresponding marker genes for fat differentiation and muscle differentiation were detected. The protein concentration was determined using the bicinchoninic acid (BCA) method (Takara). Protein sample buffer (Takara) was added into proteins at a proportion of 1:4 and denatured at 100 °C for 10 min. Polyacrylamide gels at 5% and 12% were prepared, and 20 μg of protein samples were loaded per well. Gels were electrophoresed at 80 V for 30 min, followed by 120 V until the samples ran to the bottom of the gel. The protein samples in the gel were transferred to a polyvinylidene fluoride membrane (PVDF, Merck Millipore, Taufkirchen, Germany), and then sealed with QuickBlock Western blocking solution (Beyotime Biotechnology, Shanghai, China) for 20 min. GAPDH (rabbit anti-GAPDH, 1:10,000 Abcam, Cambridge, UK, NP_001029206.1), ACTC1 (rabbit anti-ACTC1, 1:1000, Invitrogen, CA, USA NP_001029757.1), MYOD1 (mouse anti-MYOD1, 1:1000, Abcam, Cambridge, NT, UK, NP_001035568.2), MYOG (rabbit anti-MYOG, 1:1000, Abcam, Cambridge, NT, UK, NP_001104795.1), MYHC (mouse anti-MYHC, 1:200, Abcam, Cambridge, NT, UK), MRF4 (mouse anti-MYF6, 1:100, Santa Cruz Biotechnology, Santa Cruz, CA, USA, NP_861527.1), MEF2A (rabbit anti-MEF2A, 1:1000, Abcam, Cambridge, NT, UK, NP_001077107.1), FABP4 (rabbit anti-FABP4, 1:1000, Abcam, NT, UK, NP_776739.1), PPARγ (rabbit anti-PPARγ, 1:1000, Boster, Wuhan, China, NP_851367.1), FASN (rabbit anti-FASN, 1:2000, Abcam, Cambridge, NT, UK, NP_777087.1) antibodies were added and incubated overnight at 4 °C. Then, the corresponding HRP-conjugated Goat Anti-Rabbit IgG (1:5000, Sangon Biotech, Shanghai, China), or m-IgGκ BP-HRP (1:1000, sc-516102, Santa Cruz Biotechnology, Inc, Santa Cruz, CA, USA) were added and incubated at room temperature for 1 h. Finally, the prepared chemiluminescent HRP substrate (Millipore, Danvers, MA, USA) was added to the PVDF membrane containing protein bands, and the GelDoc gel XR + imaging system (Bio-Rad, Hercules, CA, USA) was used to Chemiluminescence (CL). Observe the changes in the expression of marker proteins (myoblasts: MYOD1, MYOG, MYHC, MRF4, MEF2A; preadipocytes: FABP4, PPARγ, FASN, SCD1) at different stages of cell differentiation according to the light and shade of the band.

### 2.10. Statistical Analysis

Graphpad Prism software was used to analyze quantitative results. Image Lab software was used to analyze protein results, and Modfit software was used to perform statistical analysis on the Flow Cytometry results and quantify results. Error bars represent s.e.m. * *p* < 0.05; ** *p* < 0.01.

## 3. Results

### 3.1. Screening of Adenovirus MOI

**Figure 1:**
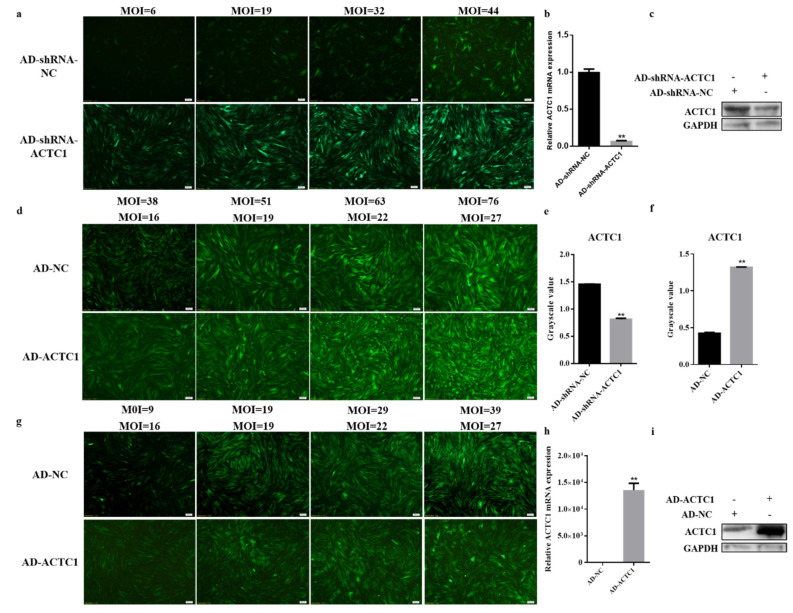
Screening of adenovirus MOI value. (**a**–**c**,**e**) Observe the expression of *ACTC1* at mRNA level and protein level and the expression of GFP after adding interfering adenovirus to myoblasts. (**e**) Use Image J software to measure the gray value of protein bands. (**d**,**f**,**h**,**i**) Observe the expression of GFP and detection of infection efficiency at mRNA level and protein level after adding expressed adenovirus to preadipocytes. (**f**) Use Image J software to measure the gray value of protein bands. (**g**) Observe the expression of GFP after adding overexpressed adenovirus to myoblasts. Each experiment was performed in triplicate. Error bars represent s.e.m. ** *p* < 0.01. Olympus IX71 microscope (40×). Original western blot figures in Figure S1h.

Figure 1a–c,e exhibits the infection of the *ACTC1* knockdown and control adenoviruses of different MOI values into myoblasts and the subsequent interference efficiency at the mRNA and protein levels. The maximal interference efficiency (up to 95%) was achieved when the AD-shRNA-NC and AD-shRNA-ACTC1 MOI values were 44 and 51, respectively. Figure 1d,f,h,i shows the infection of the *ACTC1* overexpression adenovirus into bovine preadipocytes at varying MOI levels. Maximally, at an AD-NC MOI value of 19 and an AD-ACTC1 MOI value of 9, mRNA and protein expression increased 10,000-fold compared to the control. Although *ACTC1* can be highly expressed in myoblasts, its expression cannot be detected in adipocytes; accordingly, *ACTC1* was interfered and overexpressed in myoblasts but ectopically expressed in adipocytes. Fluorescence results revealed that the optimal MOI value for overexpressing adenovirus in adipocytes was the same as in the myoblasts (Figure 1g).

### 3.2. Effect of ACTC1 on Differentiation of Bovine Myoblasts

**Figure 2:**
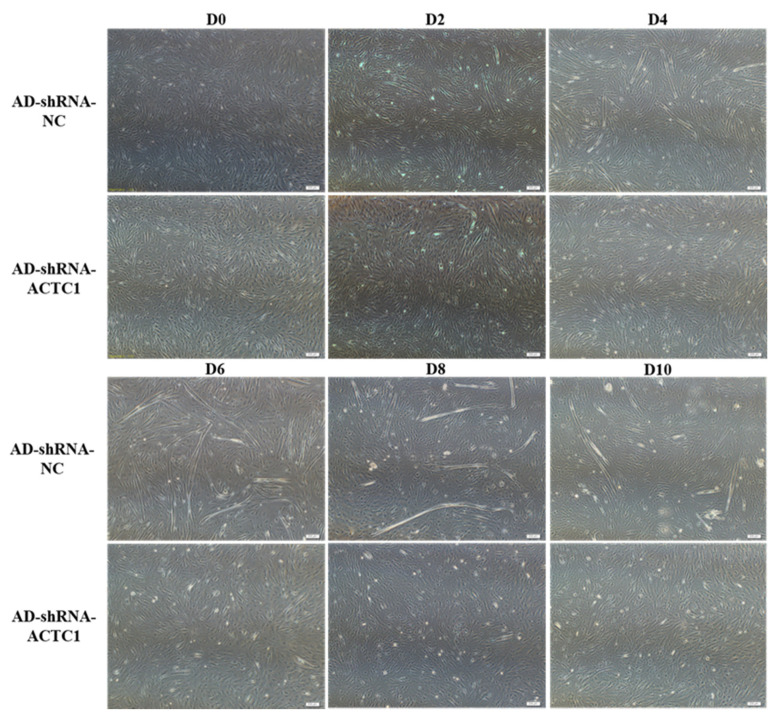
Myoblast differentiation after *ACTC1* knockdown. The myotube formation in bright field was observed 0th, 2nd, 4th, 6th, 8th, and 10th day (D0, D2, D4, D6, D8 and D10) after *ACTC1* knockdown (Olympus IX71 microscope 40×). Each experiment was performed in triplicate.

**Figure 3:**
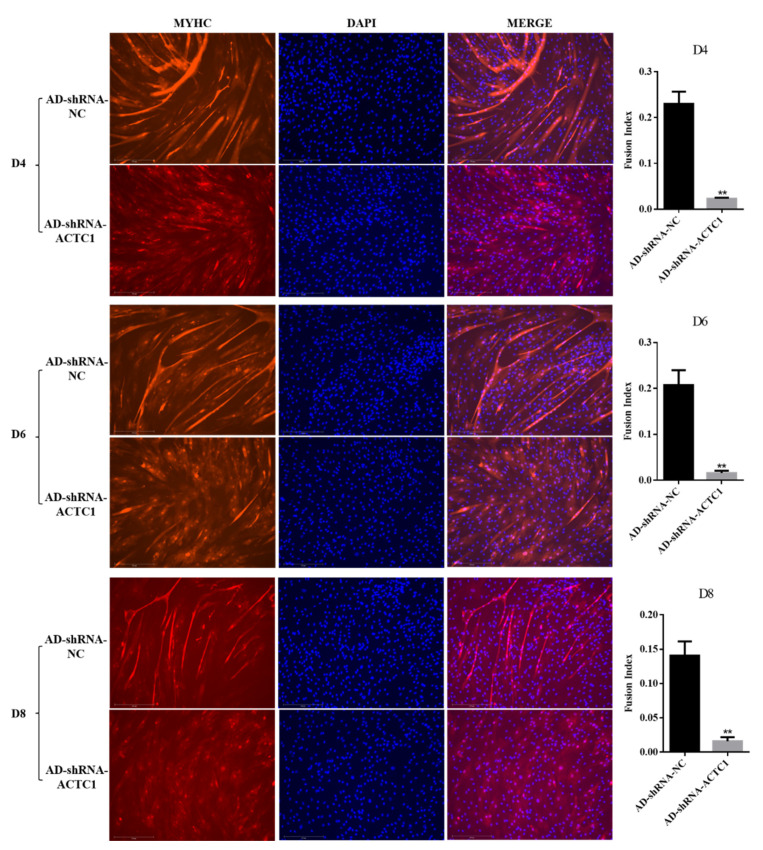
The results of MYHC immunofluorescence staining and fusion index of myoblasts on D4, D6 and D8 days after *ACTC1* knockdown (Olympus IX71 microscope 100×). Each experiment was performed in triplicate. Error bars represent s.e.m. ** *p* < 0.01.

**Figure 4:**
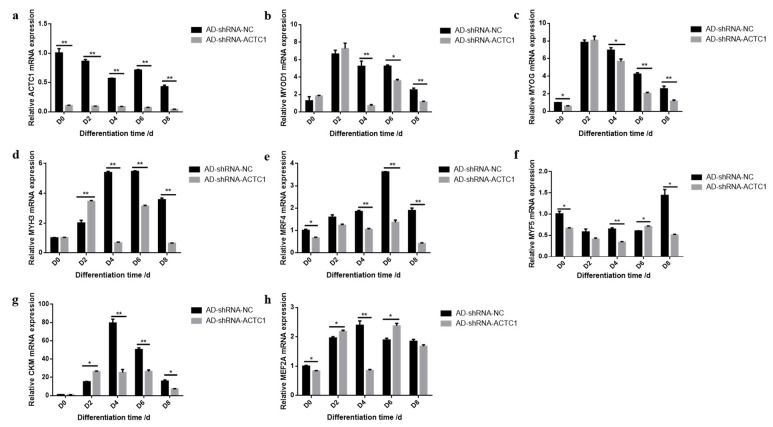
Expression of myogenic marker gene at mRNA level after *ACTC1* knockdown. (**a**–**h**) Detection of mRNA expression of *ACTC1*, *MYOD*, *MYOG*, *MYH3*, *MRF4*, *MYF5*, *CKM* and *MEF2A* on D0, D2, D4, D6, D8 days by qRT-PCR after *ACTC1* knockdown. Each experiment was performed in triplicate. Error bars represent s.e.m. * *p* < 0.05; ** *p* < 0.01.

**Figure 5:**
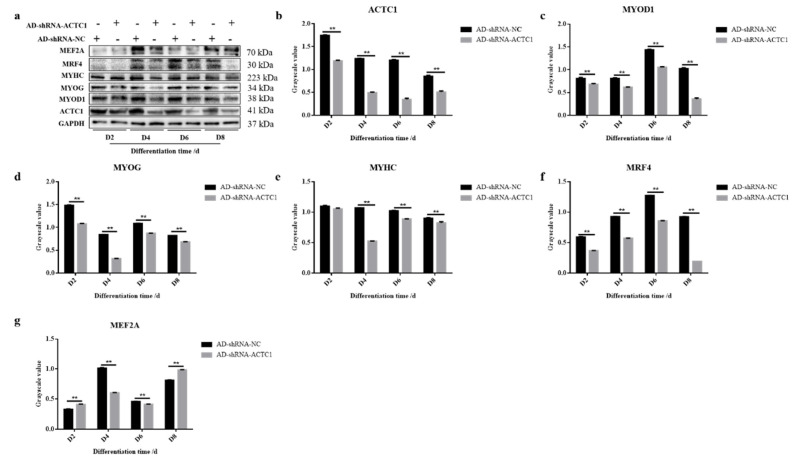
Expression of myogenic marker gene at protein level after *ACTC1* knockdown. (**a**) Detect the proteins expression of ACTC1, MYOD1, MYOG, MYHC, MRF4 and MEF2A at D2, D4, D6 and D8 days by Western blot after *ACTC1* knockdown. (**b**–**g**) Use Image J software to measure the gray value of protein bands. Each experiment was performed in triplicate. Error bars represent s.e.m. ** *p* < 0.01. Original western blot figures in Figure S1a–g.

After knockdown *ACTC1* in bovine myoblasts, the sizes and numbers of myotubes in the AD-shRNA-ACTC1 group were significantly lower than those in the AD-shRNA-NC group via brightfield imaging (D4, D6, D8, D10; Figure 2) and immunofluorescence staining (D4, D6, D8; Figure 3), with no significant differences observed at D0 or D2 (Figure 2). The myotube fusion index showed the same results (Figure 3). Myogenic marker genes, including *MYOD1*, *MYOG*, *MYH3*, *MRF4*, *MYF5* and *MEF2A*, changed significantly at the mRNA (D0, D2, D4, D6 and D8) and protein levels (D2, D4, D6 and D8) after knockdown *ACTC1* (Figure 4 and Figure 5). At the mRNA level, the expression of *MYOD1*, *MYOG*, and *MRF4* decreased significantly (*p* < 0.05 or *p* < 0.01), while the expression of *MYH3* and *CKM* increased initially at D2 (*MYH3*: *p* < 0.01; *CKM*: *p* < 0.05), then decreased significantly in the later stages (*p* < 0.05 or *p* < 0.01). *MYF5* increased significantly on D6 (*p* < 0.05 or *p* < 0.01) but decreased significantly in other periods (*p* < 0.01). The expression of MEF2A gene changed significantly depending on the day, with expression on D0 and D4 decreasing significantly (*p* < 0.01) and increasing significantly on D2 and D6 (*p* < 0.05). At protein levels, the corresponding differences can also be seen with the mRNA level (Figure 5).

**Figure 6:**
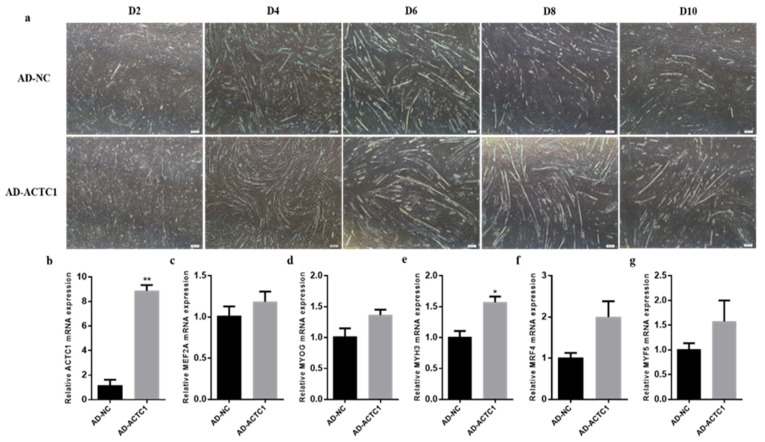
Detection of myoblast differentiation and expression of myoblast marker gene mRNA after overexpression of *ACTC1*. (**a**) The myotube formation in bright field was observed D2, D4, D6, D8 and D10 days after AD-NC and AD-ACTC1 adenovirus infection. Olympus IX71 microscope (40×); (**b**–**g**) Detection of mRNA expression of *ACTC1*, *MEF2A*, *MYOG*, *MYH3*, *MRF4* and *MYF5* on D6 after overexpression of *ACTC1*. Each experiment was performed in triplicate. Error bars represent s.e.m. * *p* < 0.05; ** *p* < 0.01.

After overexpression *ACTC1* treatment, myotube formation was observed on D2, D4, D6, D8 and D10 (Figure 6a). While there was no significant difference between AD-NC and AD-ACTC1 phenotypes on D2, the sizes and numbers of myotubes formed in the AD-ACTC1 group were significantly higher than those in AD-NC group on D4, D6, D8 and D10. The myogenic marker gene *MYH3* increased significantly (*p* < 0.05, Figure 6e) after treatment, while other marker genes showed no significant changes, although they showed an upward trend (Figure 6c,d,f,g). These results demonstrate that *ACTC1* overexpression significantly promotes myotube formation, while *ACTC1* knockdown significantly inhibits myotube formation.

### 3.3. Effect of ACTC1 on the Proliferation of Bovine Myoblasts

**Figure 7:**
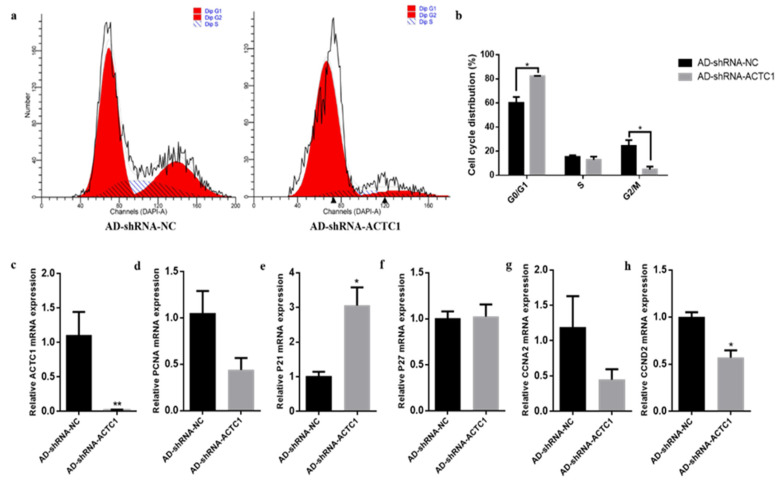
Effect of interfering *ACTC1* on proliferation cycle of bovine myoblasts and detection of mRNA expression of proliferation marker gene. (**a**,**b**) Flow cytometric measurement of DNA content using propidium iodide (PI) staining in AD-shRNA-NC/AD-shRNA-ACTC1 treated proliferating myoblast. (**c**–**h**) Relative mRNA expression of cell cycle genes: *PCNA*, *P21*, *P27*, *CCNA2* and *CCND2*. Each experiment was performed in triplicate. Error bars represent s.e.m. * *p* < 0.05; ** *p* < 0.01.

**Figure 8:**
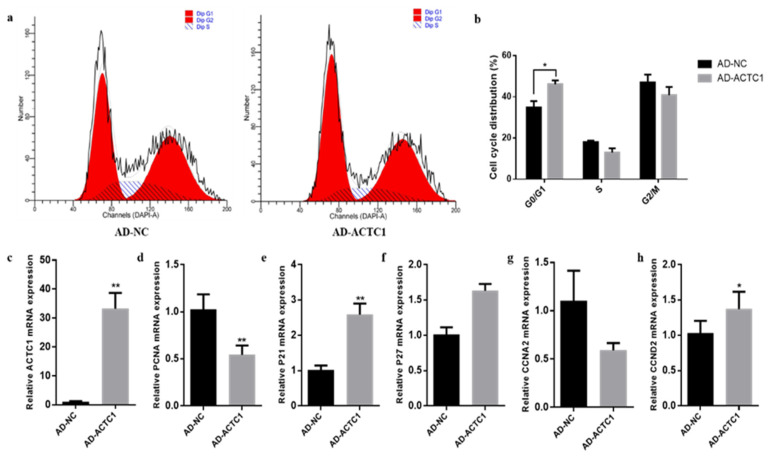
Effect of overexpression of *ACTC1* on proliferation cycle of bovine myoblasts and detection of mRNA expression of proliferation marker gene. (**a**,**b**) Flow cytometric measurement of DNA content using propidium iodide (PI) staining in AD-NC/AD-ACTC1 treated proliferating myoblast. (**c**–**h**) Relative mRNA expression of cell cycle genes: *PCNA*, *P21*, *P27*, *CCNA2* and *CCND2*. Each experiment was performed in triplicate. Error bars represent s.e.m. * *p* < 0.05; ** *p* < 0.01.

After *ACTC1* knockdown, myoblast proliferation increased significantly in the G0/G1 phase (*p* < 0.05) and decreased in G2/M phase (*p* < 0.05) (Figure 7a,b). Cell proliferation marker gene mRNA detection found that P21 increased by 201% (*p* < 0.05; Figure 6e) and *CCND2* significantly decreased (*p* < 0.05; Figure 7h), while *P27*, *PCNA* and *CCNA2* did not significantly change (Figure 7d,f,g). After *ACTC1* overexpression, the proliferation of myoblasts increased significantly in the G0/G1 phase (*p* < 0.05) but showed no significant changes in the S and G2/M phases (Figure 8a,b). Proliferation marker gene mRNA detection found that *PCNA* significantly decreased (*p* < 0.01), *P21* significantly increased (*p* < 0.01), and *CCND2* significantly increased (*p* < 0.05), while *CCNA2* and *P27* did not significantly change (Figure 8c–h). These results demonstrate that *ACTC1* can alter the proliferation of bovine myoblasts.

### 3.4. Impact of ACTC1 on Bovine Preadipocyte Differentiation

**Figure 9:**
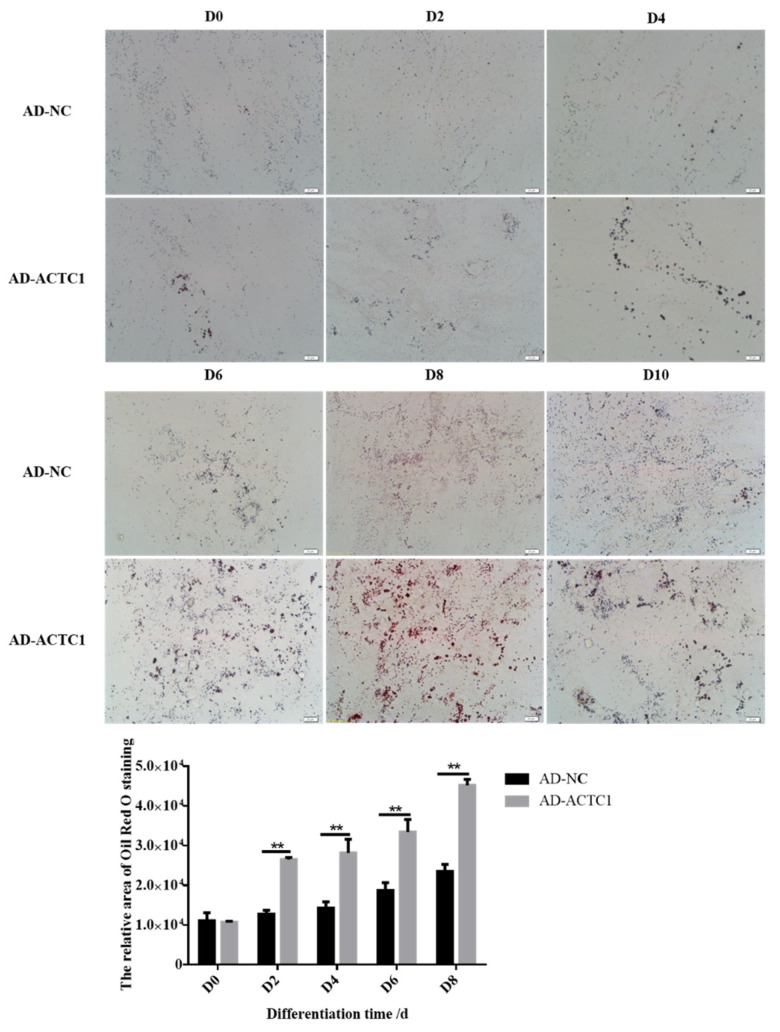
Lipid droplet formation in adipocytes after ectopic expression of *ACTC1*. Lipid droplets on D0, D2, D4, D6, D8 and D10 days after infection of AD-NC and AD-ACTC1 adenovirus by oil red O staining (Olympus IX71 microscope 400×). Each experiment was performed in triplicate. Image J software was used to measure the oil red O area. Randomly collect 3 images for each observation field. Error bars represent s.e.m. ** *p* < 0.01.

**Figure 10:**
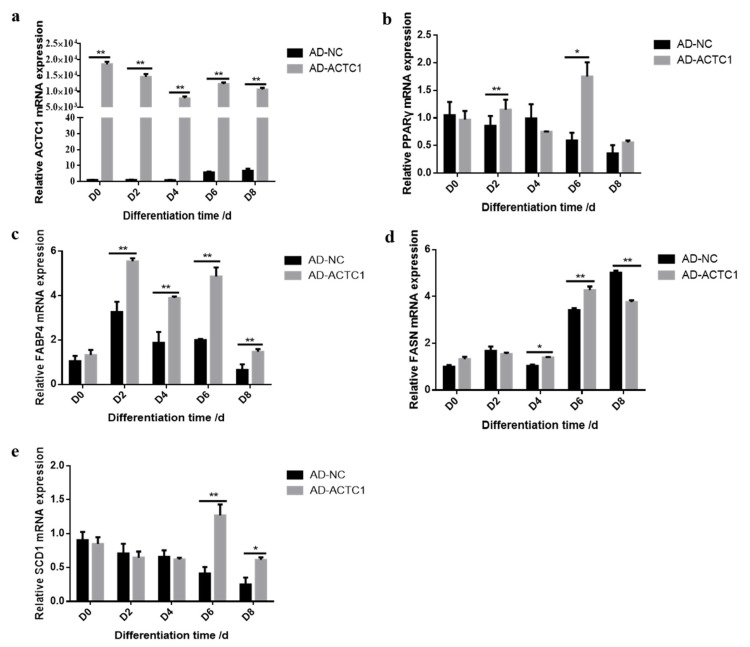
Detection of adipogenic marker gene expression at mRNA level after ectopic expression of *ACTC1*. (**a**–**e**) Detection of mRNA expression of *ACTC1*, *PPARγ*, *FABP4*, *FASN* and *SCD1* genes on D0, D2, D4, D6 and D8 days by qRT-PCR after ectopic expression *ACTC1*. Each experiment was performed in triplicate. Error bars represent s.e.m. * *p* < 0.05; ** *p* < 0.01.

**Figure 11:**
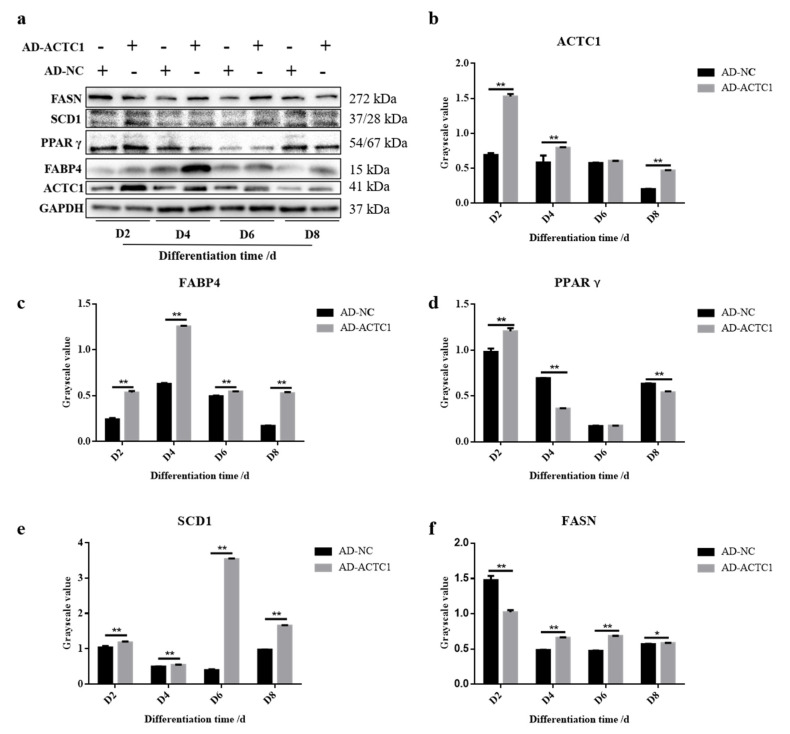
Detection of adipogenic marker gene expression at protein level after ectopic expression of *ACTC1*. (**a**) Detect the proteins expression of ACTC1, PPARγ, FABP4, FASN and SCD1 on D2, D4, D6 and D8 days by Western blot after ectopic expression of *ACTC1*. (**b**–**f**) Use Image J software to measure the gray value of protein bands. Each experiment was performed in triplicate. Error bars represent s.e.m. * *p* < 0.05; ** *p* < 0.01. Original western blot figures in Figure S2.

Oil red O staining results showed that the numbers and sizes of lipid droplets in the AD-ACTC1 group were significantly greater than those in the AD-NC group on D0, D2, D4, D6, D8 and D10 after induced ectopic *ACTC1* expression (Figure 9). Moreover, lipid quantification results showed that, except for D0, there are extremely significant differences (Figure 10). The expression of adipogenic marker genes (*PPAR γ*, *FABP4*, *FASN* and *SCD1*) were significantly changed at both the mRNA (D0, D2, D4, D6 and D8) after ectopic expression (Figure 10): *PPARγ* increased significantly on D2 (*p* < 0.01) and D6 (*p* < 0.05); *FABP4* increased significantly during adipocyte differentiation (*p* < 0.01); *FASN* increased significantly on D4 (*p* < 0.05) and D6 (*p* < 0.01), but decreased significantly on D8 (*p* < 0.01); and *SCD1* increased significantly in the late stage of adipocyte differentiation (*p* < 0.01 or *p* < 0.05; Figure 10e). The results exhibit that ectopic expression of *ACTC1* can significantly promote the formation of lipid droplets. At protein levels (D2, D4, D6 and D8), the corresponding differences with the mRNA level can also be seen (Figure 11).

### 3.5. Effect of ACTC1 on Proliferation of Bovine Preadipocytes

**Figure 12:**
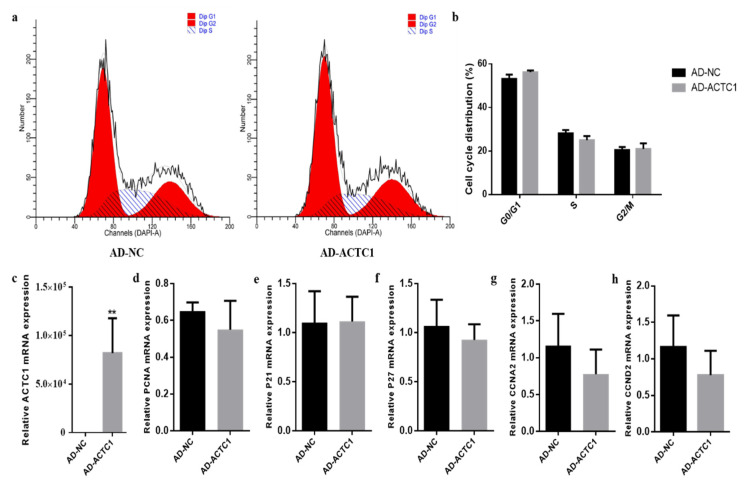
Effect of ectopic expression of *ACTC1* on proliferation cycle of bovine adipocytes and detection of mRNA expression of proliferation marker gene. (**a**,**b**) Flow cytometric measurement of DNA content using propidium iodide (PI) staining in AD-NC/AD-ACTC1 treated proliferating myoblast. (**c**–**h**) Relative mRNA expression of cell cycle genes: *PCNA*, *P21*, *P27*, *CCNA2* and *CCND2*. Each experiment was performed in triplicate. Error bars represent s.e.m. ** *p* < 0.01.

Ectopic expression of *ACTC1* had no significant effect on the proliferation cycle of preadipocytes (Figure 12a,b) and induced no significant changes in the expression of proliferation the marker genes: *PCNA*, *P21*, *P27*, *CCNA2* and *CCND2* [29,30,31,32,33,34,35] (Figure 12d–h). These results show that *ACTC1* gene had no effect on the proliferation of bovine preadipocytes.

### 3.6. Model Diagram

**Figure 13:**
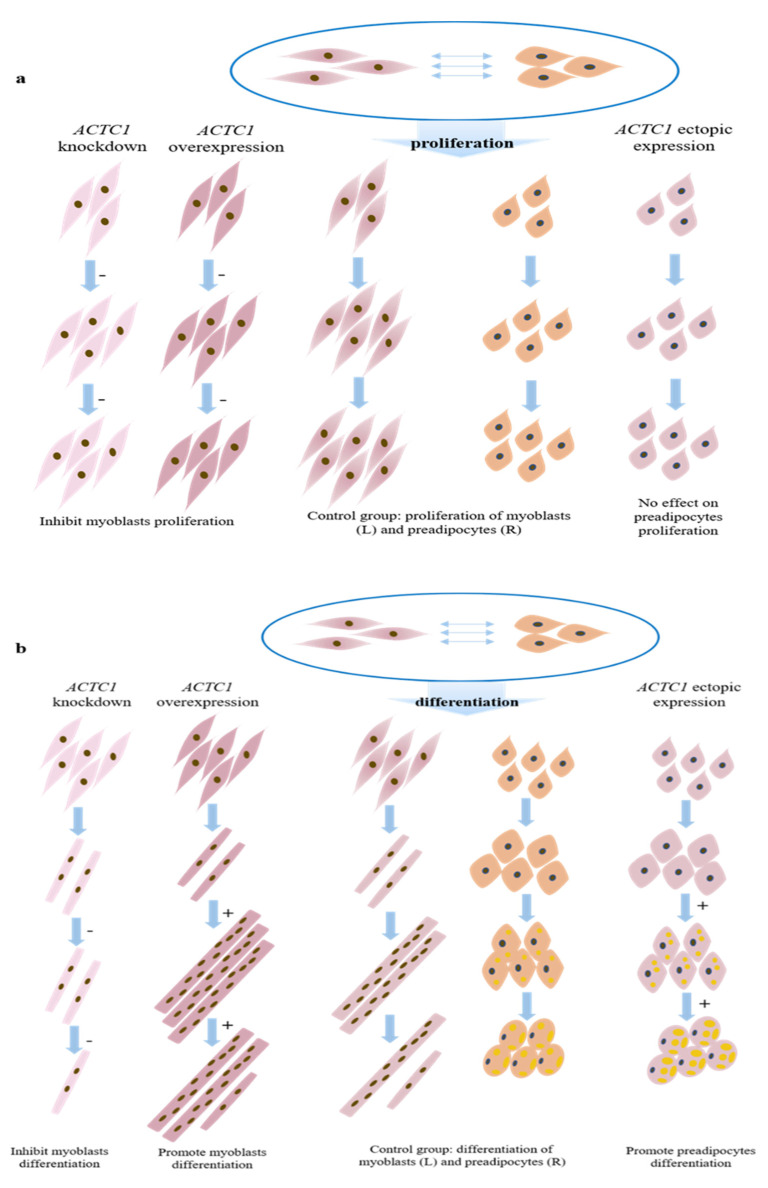
Proliferation (**a**) and differentiation (**b**) model of myoblasts and preadipocytes. 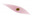 and 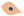 respectively represent myoblasts and preadipocytes (all infected with control adenovirus); 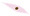 represents the myoblasts after knocking down *ACTC1*; 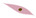 represents the myoblasts after overexpression of *ACTC1*; 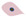 are preadipocytes after ectopic expression *ACTC1*; “−” means inhibition; and “+” means promotion.

In this study, knockdown and overexpression of *ACTC1* inhibited myoblast proliferation, and ectopic expression of *ACTC1* had no significant effect on the proliferation of bovine preadipocytes (Figure 13a). However, *ACTC1* is positively correlated with myoblast differentiation, especially having a significant promoting effect on myotube formation in the middle stage of differentiation and a significant maintenance effect on myotube in the later stage of differentiation; in addition, ectopic expression *ACTC1* could significantly promote the formation of lipid droplets, which was positively correlated with preadipocyte differentiation (Figure 13b), and this was consistent with the results of myoblasts inducing the expression of *ACTC1* in preadipocytes in the previous muscle-fat dialogue.

## 4. Discussion

The growth and development of skeletal muscle can be divided into three stages: embryonic, fetal, and mature [36]. Prenatal development is divided into two stages: embryonic and fetal skeletal muscle development. The development of skeletal muscle includes myogenesis, adipogenesis, and fibrogenesis. There are numerous factors that affect myogenesis. Genes such as *PAX3*, *PAX7*, *MYF5* and *MYOD* primarily play roles in muscle satellite cell proliferation [37,38], while *MYOD*, *MYOG*, *MRF4*, *MYHC* and *CKM* genes mainly regulate muscle cell fusion [39]. Preadipocytes are the basic biological unit of adipose tissue, and their ability to proliferate and differentiate continues throughout an animal’s life [40]. Fat differentiation depends primarily on cascade regulation of a variety of signal transduction pathways and transcription factors. Studies have shown that factors such as *PPARγ*, *SCD1*, *FABP4* and *FASN* can promote adipogenesis [41,42,43,44,45]. Since intramuscular fat content is a critical aspect of beef quality [1], and that at the cellular level, the proliferation and differentiation of myoblasts and preadipocytes directly determines the growth and development of muscle, these factors directly affect beef quality.

In a previous study, we screened the differential gene *ACTC1* via transcriptome sequencing technology. Our prior study found that *ACTC1* was highly expressed in myoblasts but was undetectable in adipocytes. In the present study, *ACTC1* was interfered and overexpressed in myoblasts, and ectopically expressed in adipocytes, to further investigate this phenomenon. First, we evaluated the impact of different titers of adenovirus—as evaluated by MOI—and determined the optimal adenovirus quantities to be added, according to myoblasts and preadipocytes post-infection status and mRNA and protein levels. Fluorescence imaging revealed that the cellular states of preadipocytes and myoblasts treated with the overexpression adenovirus were highly analogous, with the optimal MOI value for the overexpression adenovirus remaining consistent between myoblasts and preadipocytes.

Our results demonstrate that *ACTC1* knockdown in myoblasts significantly inhibits the formation of myotubes in the middle and late differentiation stages, and that *ACTC1* overexpression can promote myogenesis, most notably for the maintenance of myotubes in the later stages of differentiation. Regardless, this study exhibits that *ACTC1* can be positively correlated with myogenesis, which is consistent with existing reports [46]. Furthermore, when investigating the expression of myogenic factors, *MEF2A* was found to undergo significant changes. The *MEF2A* expression on D0 and D4 decreased significantly, but increased significantly on D2 and D6. Notably, *MEF2A* itself does not have myogenic activity, but it can directly bind to the promoters or enhancers of most muscle-specific genes and potentially assist through transcriptional cooperation [47,48].

In cell proliferation, the interaction between cyclin and cyclin-dependent kinase (CDK) strictly controls the cell cycle. The P21 family of proteins bind to cyclins, thus preventing CDK phosphorylation and consequently inhibiting the cell cycle [29,32]. In myoblasts, after *ACTC1* knockdown, the G0/G1 phase increased significantly, the G2 phase decreased significantly, and there was no significant change in the S phase. Additionally, *P21* expression increased significantly, while *CCND2* decreased significantly. After *ACTC1* overexpression, the G0/G1 phase also increased significantly, but there were no significant changes to either the G2/M phase or the S phase. In addition, *PCNA* expression significantly decreased, while *P21* and *CCND2* expression significantly increased. These results indicate that both the knockdown and the overexpression of *ACTC1* inhibited myoblast proliferation. As indicated above, a potential explanation for this phenomenon is the high background expression of *ACTC1* in muscle cells. Consequently, the overexpression of *ACTC1* may lead to cell cycle regulation disorder, causing an excess to inhibit proliferation as well. Either way, the mechanism by which *ACTC1* is involved in the regulation of myoblast proliferation cycle is complex and needs further study. Through this study, the effects of *ACTC1* on the proliferation and differentiation of bovine myoblasts were clarified for the first time.

Ectopic expression of *ACTC1* in preadipocytes demonstrated that *ACTC1* could significantly promote the formation of lipid droplets and that the expression of lipid production-related factors increases significantly with ectopic expression. However, it was also found that the ectopic expression of *ACTC1* had no significant effect on the cell cycle of preadipocytes. These findings indicate that although *ACTC1* is not expressed in adipocytes cultured in vitro, but in the real environment of marbled beef in vivo, myocytes can induce its expression and promote adipogenesis. However, since this is the first study of this gene on bovine preadipocytes and it was the first time that *ACTC1* was found to promote the differentiation of adipocytes, there are few papers that can be used as references to elucidate *ACTC1*’s specific regulatory mechanism, and it will have to be determined in future studies.

## 5. Conclusions

Herein, using the knockdown, overexpression, and ectopic expression of *ACTC1*, we found that *ACTC1* was positively correlated to the differentiation of bovine myoblasts and preadipocytes. Although *ACTC1* was found not to have a significant effect on the proliferation of preadipocytes, it could affect the proliferation of myoblasts, and its specific mechanisms require further investigation. In this study, the function of *ACTC1* in bovine preadipocytes and myoblasts was elucidated to lay a molecular foundation for the exploration of intramuscular fat formation and ultimately, the improvement of beef quality.

## Figures and Tables

**Table 1:** The primer sequence of the CDS region of *ACTC1*.

Gene Name	Accession Numbers	Primer Sequence (5′ > 3′)	Fragments Size (bp)
*ACTC1*	NM_001034585.2	Forward: ATGTGCGACGATGAGGAGAC Reverse: TTAGAAGCACTTGCGGTGGA	1134

**Table 2:** RT-qPCR primer sequence.

Gene Name	Accession Numbers	Primer Sequence (5′ > 3′)	Primer Length (bp)
*GAPDH*	NM_001034034	Forward: AGTTCAACGGCACAGTCAAGG Reverse: ACCACATACTCAGCACCAGCA	124
*ACTC1*	NM_001034585.2	Forward: CAGTGCTGTCCCTGTATGCT Reverse: AAGCAGCCGTAGCCATTTCA	282
*PPARγ*	NM_181024	Forward: TGAAGAGCCTTCCAACTCCC Reverse: GTCCTCCGGAAGAAACCCTTG	117
*SCD1*	NM_173959	Forward: TCCGACCTAAGAGCCGAGAA Reverse: TGGGCAGCACTATTCACCAG	200
*FASN*	NM_001012669	Forward: GGCAAACGGAAAAACGGTGA Reverse: CTTGGTATTCCGGGTCCGAG	183
*FABP4*	NM_174314	Forward: TGAGATTTCCTTCAAATTGGG Reverse: CTTGTACCAGAGCACCTTCATC	101
*MYOD1*	NM_001040478	Forward: AACCCCAACCCGATTTACC Reverse: CACAACAGTTCCTTCGCCTCT	196
*MYOG*	NM_001111325	Forward: GGCGTGTAAGGTGTGTAAG Reverse: CTTCTTGAGTCTGCGCTTCT	85
*MYH3*	NM_001101835.1	Forward: AAATGAGGGATGACCGCCTG Reverse: GCACTCTTGAGAAGGGGCTT	205
*MYF5*	NM_174116	Forward: CCTCTAGTTCCAGGCTCATCTA Reverse: ACCTCCTTCCTCCTGTGTAATA	90
*MRF4*	NM_181811	Forward: GTGATAACTGCCAAGGAAGGAG Reverse: CGAGGAAATGCTGTCCACGA	93
*MEF2A*	NM_001083638	Forward: AATGAACCTCACGAAAGCAGAAC Reverse: TTAGCACATAGGAAGTATCAGGGTC	106
*CKM*	NM_174773	Forward: GTGGCTGGTGATGAGGAGTC Reverse: TTTCCCCTTGAACTCACCCG	270
*PCNA*	NM_001034494.1	Forward: TGAACCTCACCAGCATGTCCAAAAT Reverse: TTCAAATACTAGTGCCAACGTGTCCG	104
*P21*	NM_001098958.2	Forward: GACCAGCATGACAGATTTCTACCA Reverse: TGAAGGCCCAAGGCAAAAG	144
*P27*	NM_001271625.3	Forward: AGATGTCAAACGTGCGAGTG Reverse: GCCAAAGAGGTTTCTGCAAG	104
*CCND2*	NM_001076372.1	Forward: GGGCAAGTTGAAATGGAA Reverse: TCATCGACGGCGGGTAC	173
*CCNA2*	NM_001075123.1	Forward: GCCACTGGCACCTCTTGATTA Reverse: TCCACGAGGATAGCCCTCATA	231

Note: GAPDH, glyceraldehyde-3-phosphate dehydrogenase; ACTC1, actin alpha cardiac muscle 1; PPARγ, peroxisome proliferator-activated receptor γ; SCD1, stearoyl-CoA desaturase; FASN, fatty acid synthase; FABP4, fatty acid-binding proteins 4; MYOD1, myogenic differentiation 1; MYOG, myogenin; MYH3, myosin heavy chain 3; MYF5, myogenic factor 5; MRF4, myogenic regulatory factor 4; MEF2A, myocyte enhancer factor 2A; CKM, creatine kinase-M; MYHC, myosin heavy chains; PCNA, proliferating cell nuclear antigen; P21, cyclin-dependent kinase inhibitor 1A; P27, cyclin-dependent kinase; CCNA2, cyclin A2; CCND2, cyclin D2.

## Data Availability

The data presented in this study are available in article and Supplementary Materials here.

## Supplementary Materials

The following are available online at https://www.mdpi.com/article/10.3390/ani11123468/s1, Figure S1: Original western blot figures (a–g). Original western blot figures of Figure 5; (h) Original western blot figures of Figure 1; Figure S2. Original western blot figures of Figure 11.

## References

[1] Poleti M.D., Regitano L., Souza G., Cesar A., Simas R.C., Silva-Vignato B., Oliveira G.B., Andrade S., Cameron L.C., Coutinho L.L. (2018). Longissimus dorsi muscle label-Free quantitative proteomic reveals biological mechanisms associated with intramuscular fat deposition. J. Proteom..

[2] Wood J.D., Enser M., Fisher A.V., Nute G.R., Richardson R.I., Sheard P.R. (1999). Manipulating meat quality and composition. Proc. Nutr. Soc..

[3] McNally E., Dellefave L. (2009). Sarcomere mutations in cardiogenesis and ventricular noncompaction. Trends Cardiovasc. Med..

[4] Matsson H., Eason J., Bookwalter C.S., Klar J., Gustavsson P., Sunnegårdh J., Enell H., Jonzon A., Vikkula M., Gutierrez I. (2008). Alpha-Cardiac actin mutations produce atrial septal defects. Hum. Mol. Genet..

[5] Suurmeijer A.J., Clément S., Francesconi A., Bocchi L., Angelini A., Van Veldhuisen D.J., Spagnoli L.G., Gabbiani G., Orlandi A. (2003). Alpha-actin isoform distribution in normal and failing human heart: A morphological, morphometric, and biochemical study. J. Pathol..

[6] Pourcel L., Buron F., Arib G., Le Fourn V., Regamey A., Bodenmann I., Girod P.A., Mermod N. (2020). Influence of cytoskeleton organization on recombinant protein expression by CHO cells. Biotechnol. Bioeng..

[7] Augière C., Mégy S., El Malti R., Boland A., El Zein L., Verrier B., Mégarbané A., Deleuze J.F., Bouvagnet P. (2015). A Novel Alpha Cardiac Actin (ACTC1) Mutation Mapping to a Domain in Close Contact with Myosin Heavy Chain Leads to a Variety of Congenital Heart Defects, Arrhythmia and Possibly Midline Defects. PLoS ONE.

[8] Bonne G., Carrier L., Richard P., Hainque B., Schwartz K. (1998). Familial hypertrophic cardiomyopathy: From mutations to functional defects. Circ. Res..

[9] Fokstuen S., Lyle R., Munoz A., Gehrig C., Lerch R., Perrot A., Osterziel K.J., Geier C., Beghetti M., Mach F. (2008). A DNA resequencing array for pathogenic mutation detection in hypertrophic cardiomyopathy. Hum. Mutat..

[10] Mogensen J., Klausen I.C., Pedersen A.K., Egeblad H., Bross P., Kruse T.A., Gregersen N., Hansen P.S., Baandrup U., Borglum A.D. (1999). Alpha-Cardiac actin is a novel disease gene in familial hypertrophic cardiomyopathy. J. Clin. Investig..

[11] Marian A.J., Roberts R. (1998). Familial hypertrophic cardiomyopathy: A paradigm of the cardiac hypertrophic response to injury. Ann. Med..

[12] Olson T.M., Michels V.V., Thibodeau S.N., Tai Y.S., Keating M.T. (1998). Actin mutations in dilated cardiomyopathy, a heritable form of heart failure. Science.

[13] Rangrez A.Y., Kilian L., Stiebeling K., Dittmann S., Yadav P., Schulze-Bahr E., Frey N., Frank D. (2020). Data on the role of cardiac α-actin (ACTC1) gene mutations on SRF-Signaling. Data Brief..

[14] Yang Q.L., Bian Y.Y., Wang B., Zuo L., Zhou M.Y., Shao H., Zhang Y.M., Liu L.W. (2019). Novel phenotype-Genotype correlations of hypertrophic cardiomyopathy caused by mutation in α-Actin and myosin-Binding protein genes in three unrelated Chinese families. J. Cardiol..

[15] Boutilier J.K., Taylor R.L., Ram R., McNamara E., Nguyen Q., Goullée H., Chandler D., Mehta M., Balmer L., Laing N.G. (2017). Variable cardiac α-Actin (Actc1) expression in early adult skeletal muscle correlates with promoter methylation. Biochim. Biophys. Acta Gene Regul. Mech..

[16] Vandekerckhove J., Bugaisky G., Buckingham M. (1986). Simultaneous expression of skeletal muscle and heart actin proteins in various striated muscle tissues and cells. A quantitative determination of the two actin isoforms. J. Biol. Chem..

[17] Dennis R.A., Przybyla B., Gurley C., Kortebein P.M., Simpson P., Sullivan D.H., Peterson C.A. (2008). Aging alters gene expression of growth and remodeling factors in human skeletal muscle both at rest and in response to acute resistance exercise. Physiol. Genom..

[18] Moll R., Holzhausen H.J., Mennel H.D., Kuhn C., Baumann R., Taege C., Franke W.W. (2006). The cardiac isoform of alpha-Actin in regenerating and atrophic skeletal muscle, myopathies and rhabdomyomatous tumors: An immunohistochemical study using monoclonal antibodies. Virchows Arch..

[19] Ilkovski B., Clement S., Sewry C., North K.N., Cooper S.T. (2005). Defining alpha-Skeletal and alpha-Cardiac actin expression in human heart and skeletal muscle explains the absence of cardiac involvement in ACTA1 nemaline myopathy. Neuromuscul. Disord..

[20] Moss J.B., Olson E.N., Schwartz R.J. (1996). The myogenic regulatory factor MRF4 represses the cardiac alpha-Actin promoter through a negative-Acting N-Terminal protein domain. J. Biol. Chem..

[21] Ohtaki S., Wanibuchi M., Kataoka-Sasaki Y., Sasaki M., Oka S., Noshiro S., Akiyama Y., Mikami T., Mikuni N., Kocsis J.D. (2017). ACTC1 as an invasion and prognosis marker in glioma. J. Neurosurg..

[22] Kim E.K., Song M.J., Jung Y., Lee W.S., Jang H.H. (2019). Proteomic Analysis of Primary Colon Cancer and Synchronous Solitary Liver Metastasis. Cancer Genom. Proteom..

[23] Mei C., Junjvlieke Z., Raza S., Wang H., Cheng G., Zhao C., Zhu W., Zan L. (2020). Copy number variation detection in Chinese indigenous cattle by whole genome sequencing. Genomics.

[24] Keel B.N., Zarek C.M., Keele J.W., Kuehn L.A., Snelling W.M., Oliver W.T., Freetly H.C., Lindholm-Perry A.K. (2018). RNA-Seq Meta-Analysis identifies genes in skeletal muscle associated with gain and intake across a multi-Season study of crossbred beef steers. BMC Genom..

[25] Li J., Liu J., Liu S., Campanile G., Salzano A., Gasparrini B., Plastow G., Zhang C., Wang Z., Liang A. (2020). Genome-Wide association study for buffalo mammary gland morphology. J. Dairy Res..

[26] Wang Y.N., Yang W.C., Li P.W., Wang H.B., Zhang Y.Y., Zan L. (2018). Myocyte enhancer factor 2A promotes proliferation and its inhibition attenuates myogenic differentiation via myozenin 2 in bovine skeletal muscle myoblast. PLoS ONE.

[27] Meissburger B., Perdikari A., Moest H., Müller S., Geiger M., Wolfrum C. (2016). Regulation of adipogenesis by paracrine factors from adipose stromal-Vascular fraction—A link to fat depot-Specific differences. Biochim. Biophys. Acta.

[28] Li P., Wang Y., Zhang L., Ning Y., Zan L. (2018). The Expression Pattern of PLIN2 in Differentiated Adipocytes from Qinchuan Cattle Analysis of Its Protein Structure and Interaction with CGI-58. Int. J. Mol. Sci..

[29] Chen J., Saha P., Kornbluth S., Dynlacht B.D., Dutta A. (1996). Cyclin-Binding motifs are essential for the function of p21CIP1. Mol. Cell. Biol..

[30] Deng C., Zhang P., Harper J.W., Elledge S.J., Leder P. (1995). Mice lacking p21CIP1/WAF1 undergo normal development, but are defective in G1 checkpoint control. Cell.

[31] Harashima H., Dissmeyer N., Schnittger A. (2013). Cell cycle control across the eukaryotic kingdom. Trends Cell. Biol..

[32] Haines D.S., Landers J.E., Engle L.J., George D.L. (1994). Physical and functional interaction between wild-Type p53 and mdm2 proteins. Mol. Cell. Biol..

[33] Resnitzky D., Hengst L., Reed S.I. (1995). Cyclin A-Associated kinase activity is rate limiting for entrance into S phase and is negatively regulated in G1 by p27Kip1. Mol. Cell. Biol..

[34] Toyoshima H., Hunter T. (1994). p27, a novel inhibitor of G1 cyclin-Cdk protein kinase activity, is related to p21. Cell.

[35] Xiong Y. (1996). Why are there so many CDK inhibitors?. Biochim. Biophys. Acta.

[36] Du M., Tong J., Zhao J., Underwood K.R., Zhu M., Ford S.P., Nathanielsz P.W. (2010). Fetal programming of skeletal muscle development in ruminant animals. J. Anim. Sci..

[37] Buckingham M., Rigby P.W. (2014). Gene regulatory networks and transcriptional mechanisms that control myogenesis. Dev. Cell.

[38] Dumont N.A., Bentzinger C.F., Sincennes M.C., Rudnicki M.A. (2015). Satellite Cells and Skeletal Muscle Regeneration. Compr. Physiol..

[39] Zammit P.S. (2017). Function of the myogenic regulatory factors Myf5, MyoD, Myogenin and MRF4 in skeletal muscle, satellite cells and regenerative myogenesis. Semin. Cell Dev. Biol..

[40] Rodriguez A.M., Elabd C., Delteil F., Astier J., Vernochet C., Saint-Marc P., Guesnet J., Guezennec A., Amri E.Z., Dani C. (2004). Adipocyte differentiation of multipotent cells established from human adipose tissue. Biochem. Biophys. Res. Commun..

[41] Bai Y., McCoy J.G., Levin E.J., Sobrado P., Rajashankar K.R., Fox B.G., Zhou M. (2015). X-Ray structure of a mammalian stearoyl-CoA desaturase. Nature.

[42] Boord J.B., Fazio S., Linton M.F. (2015). Cytoplasmic fatty acid-Binding proteins: Emerging roles in metabolism and atherosclerosis. Curr. Opin. Lipidol..

[43] Chawla A., Schwarz E.J., Dimaculangan D.D., Lazar M.A. (1994). Peroxisome proliferator-Activated receptor (PPAR) gamma: Adipose-Predominant expression and induction early in adipocyte differentiation. Endocrinology.

[44] Furuhashi M., Hotamisligil G.S. (2008). Fatty acid-Binding proteins: Role in metabolic diseases and potential as drug targets. Nat. Rev. Drug. Discov..

[45] Lefterova M.I., Haakonsson A.K., Lazar M.A., Mandrup S. (2014). PPARγ and the global map of adipogenesis and beyond. Trends Endocrinol. Metab..

[46] Tsao J., Vernet D.A., Gelfand R., Kovanecz I., Nolazco G., Bruhn K.W., Gonzalez-Cadavid N.F. (2013). Myostatin genetic inactivation inhibits myogenesis by muscle-Derived stem cells in vitro but not when implanted in the mdx mouse muscle. Stem. Cell Res. Ther..

[47] Black B.L., Olson E.N. (1998). Transcriptional control of muscle development by myocyte enhancer factor-2 (MEF2) proteins. Annu. Rev. Cell Dev. Biol..

[48] Molkentin J.D., Black B.L., Martin J.F., Olson E.N. (1995). Cooperative activation of muscle gene expression by MEF2 and myogenic bHLH proteins. Cell.

