# Supplementary material for: Effect of Actin Alpha Cardiac Muscle 1 on the Proliferation and Differentiation of Bovine Myoblasts and Preadipocytes
Source: Animals (Basel). 2021 Dec 6;11(12):3468. doi: 10.3390/ani11123468 (PMC8698029; doi:10.3390/ani11123468)
Supplement: Supplementary file 1 [file animals-11-03468-s001.zip › animals-1464243-supplementary.pdf]

Supplementary Materials:

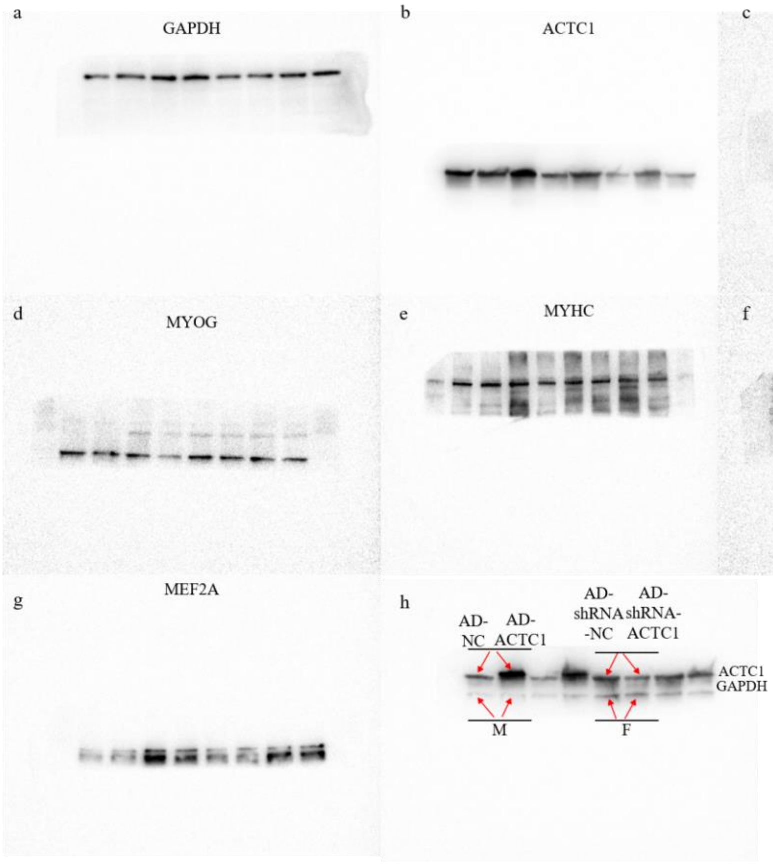

Figure S1. Original western blot figures (a–g) Original western blot figures of Figure 5; (h) Original western blot figures of Figure 1.

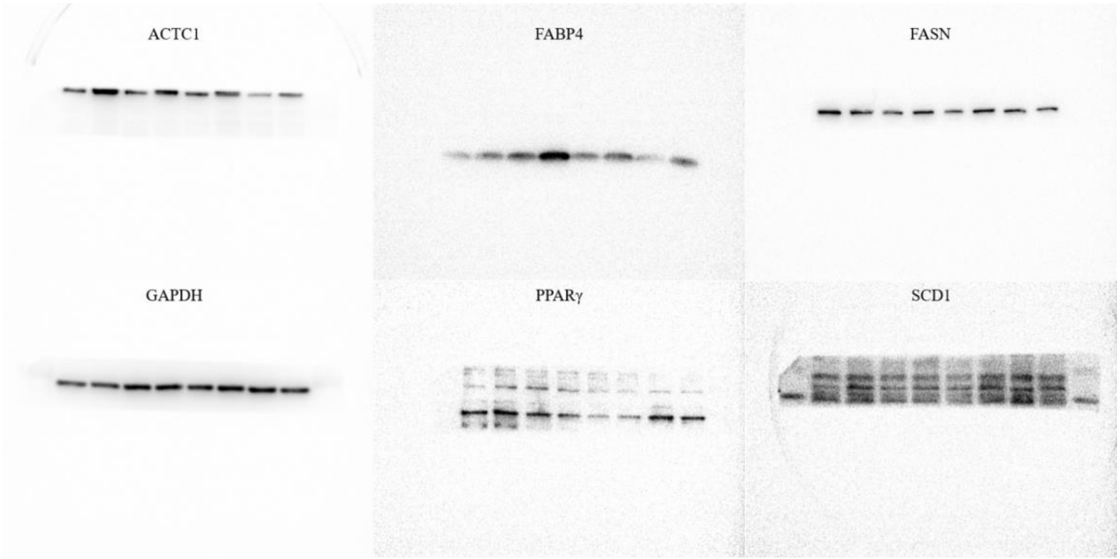

Figure S2. Original western blot figures of Figure 11.
